# Supplementary material for: Understanding the role of diabetes in the osteoarthritis disease and treatment process: a study protocol for the Swedish Osteoarthritis and Diabetes (SOAD) cohort
Source: BMJ Open. 2019 Dec 17;9(12):e032923. doi: 10.1136/bmjopen-2019-032923 (PMC6937096; doi:10.1136/bmjopen-2019-032923)
Supplement: Author's manuscript [file bmjopen-2019-032923.draft_revisions.pdf]

BMJ Open

BMJ Open is committed to open peer review. As part of this commitment we make the peer review history of every article we publish publicly available.

When an article is published we post the peer reviewers' comments and the authors' responses online. We also post the versions of the paper that were used during peer review. These are the versions that the peer review comments apply to.

The versions of the paper that follow are the versions that were submitted during the peer review process. They are not the versions of record or the final published versions. They should not be cited or distributed as the published version of this manuscript.

BMJ Open is an open access journal and the full, final, typeset and author-corrected version of record of the manuscript is available on our site with no access controls, subscription charges or pay-per-view fees (http://bmjopen.bmj.com).

If you have any questions on BMJ Open's open peer review process please email info.bmjopen@bmj.com

BMJ Open

Understanding the role of diabetes in the osteoarthritis disease and treatment process: The Swedish Osteoarthritis and Diabetes (SOAD) cohort

Journal:	*BMJ Open*
Manuscript ID	bmjopen-2019-032923
Article Type:	Protocol
Date Submitted by the Author:	12-Jul-2019
Complete List of Authors:	Dell'Isola, Andrea; Lunds University Faculty of Medicine, Department of Clinical Sciences<br>Vinblad, Johanna; The Swedish Hip Arthroplasty Register, Centre of Registers Västra Götaland; Institute of Clinical Sciences, Department of Orthopaedics, Sahlgrenska Academy, University of Gothenburg<br>Lohmander, Stefan; Lunds University Faculty of Medicine, Department of Clinical Sciences<br>Svensson, Ann-Marie; Centre of Registers in Region Västra Götaland, National Diabetes Register; University of Gothenburg, Department of Molecular and Clinical Medicine<br>Turkiewicz, Aleksandra; Lund University, Dept of Orthopedics<br>Franzén, Stefan; Centre of Registers Västra Götaland, National Diabetes Register; University of Gothenburg, Health Metrics Unit, Sahlgrenska Academy<br>Nauclér, Emma; The Swedish Hip Arthroplasty Register, Centre of Registers Västra Götaland<br>W-Dahl, A; Lund University, Department of Clinical Sciences ; The Swedish Knee Arthroplasty Register<br>Abbott, Allan; Linkoping University, Department of Medical and Health Sciences (IMH)<br>Dahlberg, L; Lunds University Faculty of Medicine, Department of Clinical Sciences<br>Rolfson , Ola ; Institute of Clinical Sciences, Department of Orthopaedics, Sahlgrenska Academy, University of Gothenburg; The Swedish Hip Arthroplasty Register, Centre of Registers Västra Götaland<br>Englund, Martin; Lund University, Dept of Orthopedics
Keywords:	osteoarthritis, General diabetes < DIABETES & ENDOCRINOLOGY, register, cohort, exercise, SURGERY

SCHOLARONE™
Manuscripts

Understanding the role of diabetes in the osteoarthritis disease and treatment process: The Swedish Osteoarthritis and Diabetes (SOAD) cohort

Dell'Isola A[1]*, Vinblad J*[2,3], Lohmander LS[1], Svensson AM[5,6], Turkiewicz A[4], Franzén S[5,7], Nauclér E[2], W-Dahl A[1,8], Abbott A[9], Dahlberg LE[1], Rolfson O[#2,3], Englund M[#,4].

* Shared first authors

# Shared senior authors

Corresponding author

Andrea Dell´Isola, Lund University, Faculty of Medicine, Department of Clinical Sciences, Orthopaedics, Lund, Sweden.
Email; andrea.dellisola@med.lu.se

1. Lund University, Faculty of Medicine, Department of Clinical Sciences Lund, Orthopaedics, Lund, Sweden.

2. The Swedish Hip Arthroplasty Register, Centre of Registers Västra Götaland, Sweden.

3. Department of Orthopedics, Institute of Clinical Science, Sahlgrenska Academy, University of Gothenburg, Sweden.

4. Lund University, Faculty of Medicine, Department of Clinical Sciences Lund, Orthopaedics, Clinical Epidemiology Unit, Lund, Sweden.

5. National Diabetes Register, Center of Registers, Gothenburg, Sweden

6. Department of Molecular and Clinical Medicine, University of Gothenburg, Gothenburg, Sweden

7. Health Metrics Unit, Sahlgrenska Academy, University of Gothenburg, Gothenburg, Sweden

8. The Swedish Knee Arthroplasty Register, Lund, Sweden

9. Department of Health, Medical and Caring Sciences, Division of Physiotherapy, Faculty of Medicine and Health Sciences, Linköping University, Linköping, Sweden

Keywords: Osteoarthritis, diabetes, register, cohort, exercise, surgery.

Word count: 2692

For peer review only

ABSTRACT (298 words)

Introduction

Osteoarthritis (OA) is the most common form of arthritis and a leading cause of disability worldwide. Metabolic comorbidities such as type 2 diabetes occur with a higher rate in people with OA than in the general population. Several factors including obesity, hyperglycemia toxicity and physical inactivity have been suggested as potential links between diabetes and OA, and have been shown to negatively impact on patients' health and quality of life. However, little is known on the role of diabetes in determining the outcome of non-surgical and surgical management of OA, and at the same time, how different OA interventions may affect diabetes control. Thus, the overall aim of this project is to explore (1) the impact of diabetes on the outcome of non-surgical and surgical OA treatments, and (2) the impact of non-surgical and surgical OA treatments on diabetes control.

Methods and analysis

The study cohort is based on prospectively ascertained register data on a national level in Sweden. Data from OA patients who received a first-line non-surgical intervention and are registered in the National Quality Register for Better Management of Patients with Osteoarthritis will be merged with data from the Swedish Knee and Hip Arthroplasty Registers and the National Diabetes Register. Additional variables regarding patients' use of prescribed drugs, comorbidities, socioeconomic status, and cause of death will be obtained through other national health and population data registers. We will thus create a cohort that will allow us to follow OA patients presenting to health care through their entire care process. The linkage will be performed on an individual level using unique personal identity numbers.

Ethics and dissemination

This study received ethical approval (2019-02570). Results from this cohort will be submitted to peer-reviewed scientific journals and reported at the leading national and international meetings in the field.

For peer review only

ARTICLE SUMMARY

Strengths and limitations of this study

- This study will use a large nationwide population-based cohort based on data from national quality registers with high coverage and completeness to explore the relationship between diabetes and osteoarthritis (OA) and their related care process.
- We will include data regarding both non-surgical and surgical treatments for patients with OA giving the possibility to capture the influence of diabetes across the whole spectrum of OA treatments.
- We will include covariate information from several national registers that will allow to account for potential confounders and effect modifiers.
- A limitation of register-based studies is that the variables available and the characteristics of the treatments provided are predetermined, i.e., it is not possible to add covariates, exposures or outcomes (not in the registers) or to modify the interventions that have been given.
- People included in the BOA, SHPR and SKAR registers received an intervention due to OA. Certain treatments are given to patients with specific characteristics, which implies that selection bias and confounding by indication may bias our estimates.

For peer review only

INTRODUCTION

Osteoarthritis (OA) is the most common form of arthritis [1]. In Sweden, more than 25% of the population aged >45 years is estimated to suffer from OA related pain symptoms and associated physical activity restrictions [2]. The average annual cost for a person affected by OA is reported to exceed €2,000, while the total European expense directly attributable to OA is estimated to be as high as €700 billion [3].

In addition to the already huge health and societal burden of OA, recent studies suggest that OA patients are twice as likely to have comorbidities compared to controls of the same age, indicating that the co-occurrence of multiple conditions in OA patients is the norm rather than the exception [4]. For instance, based on data showing a higher incidence of OA in overweight patients with metabolic disorders, a metabolic OA phenotype has been hypothesised [5-7]. Among the metabolic disorders, diabetes seems to play a central role due to its high prevalence and the toxic effect that hyperglycaemia has on the cartilage and its cells, and to the motor and sensory system through peripheral neuropathy [8-10].

According to data from the Swedish National Diabetes Register (NDR), approximately 5% of the Swedish population has diabetes, with type 2 diabetes accounting for approximately 90% of the cases[11]. Persons with diabetes have a higher risk of developing cardiovascular diseases and have a 2- to 5-fold increased risk of mortality compared with the general population [12].

In OA patients the prevalence of diabetes has been reported to be nearly three times higher than in the general population [7]. Obesity is a shared risk factor for OA and diabetes and can partially explain the association between these diseases [13, 14]. In addition to the mechanical overload caused by the excess weight, adipocytes release cytokines into the bloodstream promoting chronic low-grade inflammation and activating proteolytic enzymes which can trigger matrix degradation and initiate OA. At the same time, adipose-induced low-grade inflammation influences the metabolic dysregulation underlying several metabolic disorders among others, diabetes type II [14].

Once diabetes is initiated, it further promotes cartilage degeneration and joint inflammation causing enrichment of advanced glycation endproducts (AGEs) and matrix stiffening preventing optimal cushioning of the joint [7, 15]. This process leads to a worsening in OA symptoms promoting physical inactivity and weight gain and creating a vicious cycle which maintains the metabolic dysregulation and increases joint symptoms [13, 16-18] .

For peer review only

The evidence-based first-line management for people with OA includes education and exercise, which are recommended regardless of OA disease severity [19]. Metabolic comorbidities may have a significant impact on the treatment, partially explaining the lack of response experienced by some patients.

Replacement of the knee and of the hip is an effective treatment for patients with severe OA who do not sufficiently improve after non-surgical management [20]. Due to the rising prevalence of OA and the growing demand for this procedure, the number of hip and knee replacements has dramatically increased. In Sweden (total population 10 million), 14,700 primary total hip replacements (THRs) and nearly 14,000 total knee replacements (TKRs) were performed in 2017 with OA as indication. These figures account for 81% and 97% of the annual hip and knee replacements respectively, and translates in an annual incidence of nearly 150 procedures per 100,000 persons for both THR and TKR [21, 22].

Considering the association between diabetes and OA, surprisingly little is known regarding the influence that diabetes has on the outcome of OA treatments (both non-surgical and surgical) [23, 24]. In addition, no evidence exists regarding the effect that OA treatments (both non-surgical and surgical) may have on diabetes control. Thus, merging data from multiple Swedish registers will allow us to follow patients with OA through the progress of their disease to understand how diabetes influences the OA disease process. This study cohort is created to increase knowledge of the influence that diabetes has on the outcomes of OA patients who have received non-surgical and/or surgical treatments for hip and knee OA, and the influence OA and its treatments have on the diabetes control.

For peer review only

METHODS AND ANALYSIS

Research questions

In order to understand how the coexistence of OA and diabetes influences the treatment effects in these diseases, a series of research questions have been posed. The research questions cover two main thematic areas: (1) the impact of diabetes on the outcome of non-surgical and surgical OA treatments, and (2) the impact of OA non-surgical and surgical OA treatments on diabetes control.

<u>Area 1</u>

1. What is the prevalence of diabetes in people with OA undergoing a non-surgical intervention?
2. Is the presence of diabetes, diabetes-related factors (e.g. type of diabetes, diabetes-related medication, blood pressure, HbA1c) associated with OA severity (e.g. pain intensity, pain

frequency, walking difficulties) of people with OA undergoing a self-management non-surgical intervention?

3. Is the presence of diabetes and diabetes-related factors associated with the outcomes of a self-management non-surgical intervention for people with OA (e.g. change in pain levels, pain frequency, walking difficulties)?
4. Is the presence of diabetes and diabetes-related factors associated with the risk of joint replacement in people with OA who underwent a self-management non-surgical intervention?
5. What is the incidence of reoperations and other adverse events such as thromboembolism, cardiovascular events and mortality following primary total hip or knee replacement due to OA in people with or without diabetes?
6. What diabetes-related factors are associated with the risk of reoperation and other adverse events following primary total hip or knee replacement among person with diabetes?

Area 2

7. How does a self-management non-surgical intervention for OA influence diabetes control (e.g. change in diabetes drug intake, change in Hb A1c levels) compared to comparable people with diabetes who had not taken part in the intervention?
8. How does primary total hip or knee replacement influence the diabetes control compared to comparable persons with diabetes but with no history of hip or knee arthroplasty?
9. What diabetes-related risk factors are associated with diabetes control following primary total hip or knee replacement due to OA?

Main exposures and outcomes

The exposure and outcome measures are described in table 5. Potential confounding factors for main analysis and disease sub analysis are described with examples.

INSERT TABLE 5 HERE

The Swedish OA and Diabetes cohort (SOAD)

This nationwide observational study cohort (SOAD) will be based on prospectively obtained individual-level data from four main sources: the National Quality Register for Better Management of Patients with Osteoarthritis (BOA) Register, the Swedish Hip Arthroplasty Register (SHAR), the Swedish Knee Arthroplasty Register (SKAR), and the Swedish National Diabetes Register (NDR). Data starting from the

For peer review only

year of each register establishment will be merged using the unique personal identity number (PIN) issued to all legal residents in Sweden. Additional variables regarding patients' use of prescribed drugs, comorbidities, cause of death, and socioeconomic information will be obtained through the following population-based registers:

- The Swedish Prescribed Drug Register (SPDR) held by the National Board of Health and Welfare.
- The National Patient Register (NPR) held by the National Board of Health and Welfare; information regarding in-hospital diagnoses and outpatient specialist care diagnoses, e.g. interventions, adverse events such as thromboembolism or other comorbid conditions.
- Swedish Cancer Register, The National Board of Health and Welfare.
- The Cause of death Register (CODR) held by the National Board of Health and Welfare
- Longitudinal integration database for health insurance and labour market studies (LISA) held by Statistics Sweden for data such as marital status, educational level and country of origin.

Data sources

BOA: The BOA register was started in 2008 and currently includes more than 100,000 individuals with OA of the knee or of the hip who have registered for an evidence-based self-management programme. These patients sought treatment for knee and/or hip pain in primary healthcare in Sweden and were referred for standardised core treatment (education and supervised exercises) after a confirmed clinical/radiographic OA diagnosis in accordance with the recommendations for OA diagnosis from the Swedish National Board of Health and Welfare[25]. These guidelines are in line with internationally accepted diagnostic criteria, and according to the guidelines, radiographic examination should only be used in uncertain cases, if the patient is not responding to treatment or when a surgical intervention is planned [26, 27]. BOA offers all the patients two education sessions focusing on the pathophysiology of OA and the benefit of exercise. A face-to-face session with a physiotherapist is also offered. In this session, the patients receive a personalised intervention programme and the necessary instructions to perform it independently at home. In addition, BOA offers patients the possibility to undertake up to 12 supervised group exercise session with a physical therapist. Thus, the register contains two separate cohorts that performed, in addition to the education sessions, either home exercise or supervised exercise. The register has a data completeness of almost 90% and the BOA participants have answered validated and patient-relevant socio-demographic and outcome questionnaires after the interventions (2-5 months) and at 1 year (12-15 months) (Table 1).

For peer review only

INSERT TABLE 1 HERE

SHAR: Started in 1979, SHAR registers primary hip replacement operations and reoperations in Sweden including individual patient data, surgical technique and type of implant used. Since 2002 patient-reported measures such as joint pain, HRQoL and satisfaction with treatment have also been collected before surgery and 1, 6 and 10 years postoperatively. The register encompasses 318 000 primary total hip replacements due to OA and 61 500 reoperations after total hip replacements where OA was the main reason for the primary surgery (at the end of 2018). The register has an overall data completeness of 98,5% (2016) including all indications for total hip replacements (Table 2).

SKAR: The Swedish Knee Arthroplasty Register is a Swedish National Quality Register founded in 1975. The register collects individual patient data, surgical technique and type of implant used for patients who undergo knee replacement. The SKAR also collects information on re-operations/revision surgery. SKAR has a completeness of 98.1 % (2016) and have registered almost 270,000 primary knee replacements due to OA and more than 21 400 revisions at the end of 2018 (Table 3).

INSERT TABLE 2 AND 3 HERE

NDR: NDR has been a Swedish National Quality Register since 1996 and collects data on clinical characteristics, risk factors, laboratory analyses, complications of diabetes, and medications for patients 18 years of age or older with a diagnosis of diabetes (Table 4). The completeness is 96.5 % (2017) and the register has 750 004 (2017) unique individuals in their database. More than 95% of all individuals with type 1 diabetes mellitus (T1DM) and 90% of individuals with type 2 diabetes mellitus (T2DM) in Sweden are included in the NDR.

INSERT TABLE 4 HERE

Data linkage

Personal Identity Number (PIN): In Sweden, all legal residents are registered with a unique PIN that provides information on date of birth and sex. Swedish law requires all documentation regarding healthcare contacts to be registered using the patient's PIN[28]. The PIN is also used for registration of data for statistics such as national population-based registers and healthcare quality registers [29, 30]. The system allows for linkage of data at an individual level between the different registers in Sweden with the possibility of creating merged research databases for epidemiological research on large populations,

For peer review only

after the relevant ethical approval has been obtained. Data linkage for the current study is described in figure 1.

INSERT HERE FIGURE 1

Analysis plan

We will develop a specific statistical analysis plan for each specific study that will be conducted within SOAD. These will follow a number of general principles. We will aim for inclusion of all available OA and diabetes patients to limit potential selection bias. We will use multiple imputation methods to impute the missing data on exposures, outcomes and confounders, when relevant. The imputation model will be specific for each study and compatible with the chosen analysis model. For example, the fully conditional specification (also called chained equations) may be used to enable flexible models for proper imputation of all variables. In the statistical modelling we will aim for estimation of causal effects and statistical models will be chosen accordingly using direct acyclic graphs (DAGs) to enable proper confounding control [31]. For confounding control, we will use regression models or inverse probability weighting. For analysis of panel data (i.e. longitudinal repeated measurements of the participants and/or data clustered by caregiver) we will use multilevel regression models. For time-to-event data we will use the proportional hazards Cox regression model, or, if appropriate, parametric models. For mediation analysis, we will use linear models or maximum likelihood structural equation models when appropriate. For categorical outcomes we will use other approaches [32, 33]. We will report the results from all analyses as relevant estimated effect size (such as risk differences, risk ratios of hazard ratios) with 95% confidence intervals and interpret these for clinical relevance, irrespectively of statistical significance [34, 35].

Considering the variety of exposures and outcome studied, we have not performed any specific sample size analysis. However, we will include all the available data, with ~100,000 BOA participants, whereof an estimated 15,000 have diabetes [36]. Regarding the SHAR and SKAR, we will have data for nearly 30,000 joint replacements. Based on previous studies we expect that the prevalence of diabetes will be around 8% and 14% among patients undergoing THR and TKR, respectively [37, 38]. We expect that this will enable precise estimation of the main effects of interest.

Patient and public involvement

Patient representatives were not involved in the development of the research question or the design of this study. Patients were however involved in the creation of the BOA supported self-management programme and contributed with the key content of the programme and the mode of delivery from the

For peer review only - http://bmjopen.bmj.com/site/about/guidelines.xhtml

patients' perspective [39]. The Swedish Hip and Knee Arthroplasty Registers have patient representatives on their respective steering committees.

ETHICS AND DISSEMINATION

Storage and management of data

A copy of the full data set will be stored at the Center of Registers Västra Götaland, Gothenburg, Sweden. A second copy of the full data set will be stored at Lund University on the platform LUSEC (Lund information security platform). The platforms are designed to securely store, manage and analyse data in accordance with the European Union general data protection regulation (GDPR). The process of linkage, storage and management of data, the role of informed consent in register-based research and safeguarding the integrity of study participants follows the legal and ethical frameworks as described by Swedish law and ethical boards. This has been described by Ludvigsson et al [28].

Dissemination

The results from this study will be published in peer-reviewed scientific journals and will be presented at the leading national and international meetings in the field. The results will also be disseminated through annual reports published on the registers' websites.

DISCUSSION

This study cohort will provide unique insights on the relationships between diabetes and OA. By using data from the BOA, SHAR, SKAR and NDR registers we will be able to investigate the influence of diabetes on the outcome of non-surgical and surgical OA interventions as well as the effect of OA treatments on diabetes control.

To our knowledge, this will be the largest dataset combining data on OA and diabetes management. Thanks to the large sample size on national level, results arising from this study will likely have a high external validity and generalisability. However, treatment data collected in national registers are likely to be influenced by the regional differences in treatment protocols and data collection that characterise different clinical environments when compared to e.g. highly standardised clinical trials.

In conclusion, to optimise treatments for OA and diabetes and move towards a personalised-care approach, it is important to identify factors and comorbidities that may negatively influence the outcome of the interventions. The coexistence of several conditions creates a more complex disease status which

For peer review only

271 requires additional considerations and care for the patient to experience the desired benefit from the provided interventions. The SOAD cohort will help us to identify these patients with complex needs, opening a venue for the development of better treatment approaches. Ultimately, the cohort has the potential to impact on the way OA is managed when other comorbidities coexist, potentially reducing the huge burden of this disease.

For peer review only

References

1. European Medical Agency (EMA); Guideline on clinical investigation of medicinal products used in the treatment of osteoarthritis. https://www.ema.europa.eu 2010.
2. Turkiewicz A, Petersson IF, Bjork J, Hawker G, Dahlberg LE, Lohmander LS, et al. Current and future impact of osteoarthritis on health care: a population-based study with projections to year 2032. Osteoarthritis Cartilage 2014; 22: 1826-1832.
3. Salmon JH, Rat AC, Sellam J, Michel M, Eschard JP, Guillemin F, et al. Economic impact of lower-limb osteoarthritis worldwide: a systematic review of cost-of-illness studies. Osteoarthritis Cartilage 2016; 24: 1500-1508.
4. Kadam UT, Jordan K, Croft PR. Clinical comorbidity in patients with osteoarthritis: A case-control study of general practice consumers in England and Wales. Annals of the Rheumatic Diseases 2004; 63: 408-414.
5. Dell'Isola A, Allan R, Smith SL, Marreiros SS, Steultjens M. Identification of clinical phenotypes in knee osteoarthritis: a systematic review of the literature. BMC Musculoskelet Disord 2016; 17: 425.
6. Dell'Isola A, Steultjens M. Classification of patients with knee osteoarthritis in clinical phenotypes: Data from the osteoarthritis initiative. PLoS One 2018; 13: e0191045.
7. Berenbaum F. Diabetes-induced osteoarthritis: from a new paradigm to a new phenotype. Annals of the Rheumatic Diseases 2011; 70: 1354-1356.
8. Zhuo Q, Yang W, Chen J, Wang Y. Metabolic syndrome meets osteoarthritis. Nat Rev Rheumatol 2012; 8: 729-737.
9. Yoshimura N, Muraki S, Oka H, Kawaguchi H, Nakamura K, Akune T. Association of knee osteoarthritis with the accumulation of metabolic risk factors such as overweight, hypertension, dyslipidemia, and impaired glucose tolerance in Japanese men and women: the ROAD study. J Rheumatol 2011; 38: 921-930.
10. Karvonen-Gutierrez CA, Sowers MR, Heeringa SG. Sex dimorphism in the association of cardiometabolic characteristics and osteophytes-defined radiographic knee osteoarthritis among obese and non-obese adults: NHANES III. Osteoarthritis Cartilage 2012; 20: 614-621.
11. Gudbjörnsdottier S SA, Eliasson B, Eeg Olofsson K, Linder E, Samuelsson P, Miftaraj M. Nationella Diabetesregistrets årsrapport. 2017.
12. Thomas RJ, Palumbo PJ, Melton III LJ, Roger VL, Ransom J, O'brien PC, et al. Trends in the mortality burden associated with diabetes mellitus: a population-based study in Rochester, Minn, 1970-1994. Archives of Internal Medicine 2003; 163: 445-451.
13. Wluka AE, Lombard CB, Cicuttini FM. Tackling obesity in knee osteoarthritis. Nat Rev Rheumatol 2013; 9: 225-235.
14. Hotamisligil GS. Inflammation, metaflammation and immunometabolic disorders. Nature 2017; 542: 177-185.
15. Rosa SC, Goncalves J, Judas F, Mobasheri A, Lopes C, Mendes AF. Impaired glucose transporter-1 degradation and increased glucose transport and oxidative stress in response to high glucose in chondrocytes from osteoarthritic versus normal human cartilage. Arthritis Res Ther 2009; 11: R80.
16. Mobasheri A. Glucose: an energy currency and structural precursor in articular cartilage and bone with emerging roles as an extracellular signaling molecule and metabolic regulator. Front Endocrinol (Lausanne) 2012; 3: 153.
17. Berenbaum F, Wallace IJ, Lieberman DE, Felson DT. Modern-day environmental factors in the pathogenesis of osteoarthritis. Nature Reviews Rheumatology 2018.

For peer review only

18. Verzijl N, Bank RA, TeKoppele JM, DeGroot J. AGEing and osteoarthritis: a different perspective. Curr Opin Rheumatol 2003; 15: 616-622.
19. Fernandes L, Hagen KB, Bijlsma JW, Andreassen O, Christensen P, Conaghan PG, et al. EULAR recommendations for the non-pharmacological core management of hip and knee osteoarthritis. Ann Rheum Dis 2013; 72: 1125-1135.
20. Skou ST, Roos EM, Laursen MB, Rathleff MS, Arendt-Nielsen L, Rasmussen S, et al. Total knee replacement and non-surgical treatment of knee osteoarthritis: 2-year outcome from two parallel randomized controlled trials. Osteoarthritis Cartilage 2018; 26: 1170-1180.
21. Kärrholm JMM. OD, Vinblad J, Rogmark C, Rolfson O. Svenska Höftprotesregistret Årsrapport 2017. https://shpr.demo.registercentrum.se/ 2018.
22. Robertsson O W-DA, Lidgren L, Sundberg M. Årsrapport 2018 Svenska Knäprotesregistret http://www.myknee.se/ 2018.
23. Malinzak RA, Ritter MA, Berend ME, Meding JB, Olberding EM, Davis KE. Morbidly obese, diabetic, younger, and unilateral joint arthroplasty patients have elevated total joint arthroplasty infection rates. J Arthroplasty 2009; 24: 84-88.
24. Robertson F, Geddes J, Ridley D, McLeod G, Cheng KJTK. Patients with Type 2 diabetes mellitus have a worse functional outcome post knee arthroplasty: a matched cohort study. 2012; 19: 286-289.
25. Socialstyrelsen. Nationella riktlinjer för rörelseorganens sjukdomar 2012: Osteoporos, artros, inflammatoriskryggsjukdom och ankyloserande spondylit, psoriasisartrit och reumatoid artrit: Stöd för styrning och ledning. Socialstyrelsen 2012.
26. Altman R, Alarcon G, Appelrouth D, Bloch D, Borenstein D, Brandt K, et al. The American College of Rheumatology criteria for the classification and reporting of osteoarthritis of the hip. 1991; 34: 505-514.
27. Altman R, Asch E, Bloch D, Bole G, Borenstein D, Brandt K, et al. Development of criteria for the classification and reporting of osteoarthritis: Classification of osteoarthritis of the knee. Arthritis & Rheumatism 1986; 29: 1039-1049.
28. Ludvigsson JF, Otterblad-Olausson P, Pettersson BU, Ekbom A. The Swedish personal identity number: possibilities and pitfalls in healthcare and medical research. Eur J Epidemiol 2009; 24: 659-667.
29. Ludvigsson JF, Almqvist C, Bonamy AK, Ljung R, Michaelsson K, Neovius M, et al. Registers of the Swedish total population and their use in medical research. Eur J Epidemiol 2016; 31: 125-136.
30. Ludvigsson JF, Andersson E, Ekbom A, Feychting M, Kim JL, Reuterwall C, et al. External review and validation of the Swedish national inpatient register. BMC Public Health 2011; 11: 450.
31. Textor J, van der Zander B, Gilthorpe MS, Liskiewicz M, Ellison GT. Robust causal inference using directed acyclic graphs: the R package 'dagitty'. Int J Epidemiol 2016; 45: 1887-1894.
32. Breen R, Karlson KB, Holm A. Total, direct, and indirect effects in logit and probit models. Sociological Methods & Research 2013; 42: 164-191.
33. Lange T, Hansen KW, Sorensen R, Galatius S. Applied mediation analyses: a review and tutorial. Epidemiol Health 2017; 39: e2017035.
34. Amrhein V, Greenland S, McShane B. Scientists rise up against statistical significance. Nature Publishing Group 2019.
35. McShane BB, Gal D, Gelman A, Robert C, Tackett JL. Abandon Statistical Significance. The American Statistician 2019; 73: 235-245.
36. Louati K, Vidal C, Berenbaum F, Sellam J. Association between diabetes mellitus and osteoarthritis: systematic literature review and meta-analysis. RMD open 2015; 1: e000077-e000077.

For peer review only

37. Pedersen AB, Mehnert F, Johnsen SP, Sorensen HT. Risk of revision of a total hip replacement in patients with diabetes mellitus: a population-based follow up study. J Bone Joint Surg Br 2010; 92: 929-934.
38. Lenguerrand E, Beswick AD, Whitehouse MR, Wylde V, Blom AW. Outcomes following hip and knee replacement in diabetic versus nondiabetic patients and well versus poorly controlled diabetic patients: a prospective cohort study. Acta Orthop 2018; 89: 399-405.
39. Thorstensson CA, Garellick G, Rystedt H, Dahlberg LE. Better Management of Patients with Osteoarthritis: Development and Nationwide Implementation of an Evidence-Based Supported Osteoarthritis Self-Management Programme. Musculoskeletal Care 2015; 13: 67-75.

Author contribution

All authors participated in the design of the study. All authors read, provided feedback and approved the final protocol.

Funding

JV has received funding from Dr. Felix Neuberghs Foundation. ME and AT are funded by the Swedish Research Council and The Swedish Rheumatology Association.

Data sharing

All information regarding individual patients is subject to confidentiality in accordance with The Public Access to Information and Secrecy Act (SFS 2009:400) chapter 25 article 1. Therefore, we will be unable to share any data included in the SOAD cohort.

Conflict of interest

AWD is employed at the Swedish Knee Arthroplasty Register (SKAR). JV, AMS, SF, EN, OR are employed by the Centre of Registers Västra Götaland, Sweden. AWD is employed at the Swedish Knee Arthroplasty Register (SKAR). LED is the Co-founder and Chief Medical Officer of Joint Academy, a company which provides web-based non-surgical interventions for patients with hip and knee osteoarthritis. AA is employed by the Better management of OsteoArthritis register (BOA). All the other authors have nothing to disclose

Word count: max 4,000

For peer review only

Table 1. Description of the single variables collected from the Better Management of Patients with Osteoarthritis Register (BOA)

**BOA Register**		Baseline	Evaluation 3 months	Evaluation 12 months
**Variable category**	**Variable**			
Date	Date of visits	x	x	x
Patient-reported measures	Age, sex, weight, height	x		
	Smoking	x		
	Most affected joint (hip, knee or hand)	x	x	x
	Other affected joints	x	x	x
	Fear avoidance	x	x	x
	Request for surgery	x	x	x
	Walking difficulties	x	x	x
Physical activity level	Duration of physical training [a)]	x	x	x
	Duration of physical activity [b)]	x	x	x
Satisfaction	Satisfaction with treatment		x	x
Musculoskeletal comorbidity	Charnley class [c)]	x	x	x
Pain	Pain severity [d)] NRS	x	x	x
	Pain frequency	x	x	x
Generic	EQ-5D	x	x	x
Self-efficacy	Arthritis self-efficacy scale	x	x	x
Physiotherapist-reported measures	Earlier radiography/MRI/surgery in the most affected or the contralateral joint	x		
	Earlier treatments (including physiotherapy/adapted training/information on weight reduction/pharmaceuticals)	x		
	Waiting list for surgery	x	x	
	Use of medications for OA	x	x	
Follow-up	Radiography/MRI/surgery in the most affected or the contralateral joint since last evaluation		x	
	Compliance with intervention		x	

For peer review only

[a)] Answering to the question:" During a regular week, how much time do you spend exercising on a level that makes you short winded, for example running, fitness class, or ball games?" graded on categorical scale from "0" to "more than 120 minutes".
[b)] Answering to the question: "During a regular week, how much time are you physically active in ways that are not exercise, for example walks, bicycling, or gardening?" graded on categorical scale from "0" to "more than 300 minutes".[25]
NRS: Numeric Rating Scale, VAS: visual analogue scale, MRI: magnetic resonance imaging

For peer review only

409 Table 2. Description of single variables collected from the Swedish Hip Arthroplasty Register

**Swedish Hip Arthroplasty Register**		**Baseline**	**Follow-up 1, 6 and 10 year**
**Variable category**	**Variables**		
Surgery-related variables			
Diagnosis (at hip)	ICD-10	x	
	Laterality	x	
Date	Date of surgery	x	
Type of surgery	Primary, revision, other reoperation	x	
Type of replacement	Total, partial, resurfacing hip replacement	x	
Patient status	Age, sex, height , weight, ASA class	x	
Implant characteristics	Article number, type of implant	x	
Technique	Incision, fixation	x	
Patient-reported measures			
Smoking status	Smoking (never, ex, daily, not daily))	x	
Musculoskeletal comorbidity	Charnley class [a)]	x	x
		x	x
Generic	EQ-5D	x	x
Treatment before hip replacement surgery	Physiotherapy	x	
	Standardised core treatment of education and supervised exercises	x	
Disease specific	Hip pain (Likert)	x	x
Satisfaction	Satisfaction with treatment (Likert)		x

410 [a)] Charnley class: classifications of musculoskeletal impairment. Class A corresponds to unilateral hip disease; Class
411 B bilateral hip disease; and Class C indicates multiple joint disease or some other condition that inhibits the
412 patient's ability to walk.
413 ICD-10, International Classification of Diseases, tenth revision; VAS, visual analogue scale. ASA-class, American
414 Society of Anesthesiologists physical status classification system
415

For peer review only

Table 3. Description of single variables collected from the Swedish Knee Arthroplasty Register

**Swedish Knee Arthroplasty Register**		Baseline	Follow-up 1 year
**Variable category**	**Variable**		
Surgery-related variables			
Diagnosis (at knee)	ICD-10	x	
	Laterality	x	
Date	Date of surgery	x	
Type of surgery	Primary, revision	x	
Type of replacement	Total, uni-compartmental, stabilised (hinged) knee replacement	x	
Patient status	Age, sex, height, weight, ASA class	x	
Implant characteristics	Article number, type of implant	x	
Technique	Incision, fixation	x	
Patient-reported measures			
Musculoskeletal comorbidity	Charnley class (modified) [a)]	x	x
		x	x
Generic	EQ-5D	x	x
Satisfaction	Satisfaction with treatment (VAS)		x
Disease specific	Knee injury and Osteoarthritis Outcome Score (KOOS), Knee pain (VAS)	x	x

[a)] Charnley class: classifications of musculoskeletal impairment. Class A: unilateral knee disease; Class B1:bilateral OA, one knee is scheduled for or already received arthroplasty surgery while the other knee has OA but no scheduled arthroplasty surgery; B2: bilateral OA, one knee is scheduled for or already received arthroplasty surgery while the other knee has already received knee arthroplasty surgery; Class C to multiple joint disease or some other condition that inhibits the patient's ability to walk.

ICD-10, International Classification of Diseases, tenth revision; VAS, visual analogue scale. ASA-class, American Society of Anesthesiologists physical status classification system

For peer review only

Table 4. Description of single variables collected from the National Diabetes Register

National diabetes register

**Variable category**	**Variable**
Patient's characteristics	Age (years), sex, height, weight, BMI
Diabetes characteristics	Type of diabetes, HbA1c(mmol/mol), debut year of diabetes, diabetes duration (years), age at onset
Diabetes treatment	Diet only, insulin, tablets, tablets and insulin
Method of insulin delivery	Insulin Pump Treatment (CSI), multiple daily injections (MDI)
Blood pressure	Systolic blood pressure (mmHg), Diastolic blood pressure (mmHg)
Cholesterol	Total cholesterol (mmol/L), LDL (mmol/L), HDL (mmol/L)
HbA1c	Triglycerides (mmol/L)
Renal function	Creatinine (µmol/L), eGFR (mL/min/1.73 m2)
Retinopathy	Retinopathy (yes/no)
Other treatments	Anti-hypertensive treatment, Lipid-lowering treatment,
Physical Activity	Times per week of moderate to intense physical activity
Smoking status	Smoking (yes/no)
Albuminuria	Micro albuminurea, macro albuminurea (%)

Variables are measured at least once per year for patients with diabetes type 2 and four times per year for patients with diabetes type 1. If the patient has specific problems variables may be recorded with higher frequency. BMI: body mass index; HbA1: Haemoglobin subunit alpha 1; LDL: low density lipoprotein, HDL: high density lipoprotein; eGFR: estimated glomerular filtration rate

For peer review only

Table 5. Exposure and outcome for the study populations and examples of confounders and effect modifiers for the study analyses.

Population	Exposure	Outcome	Example confounders and effect modifiers
**People with OA**			
**undergoing self-management treatment**	diabetes	• Pain intensity<br>• Pain frequency<br>• Walking difficulties<br>• Quality of life<br>• Use of pain medications<br>• Sick leave	• Patient characteristics (age, sex, BMI, smoking)<br>• Type of diabetes<br>• Diabetes medications<br>• Diabetes severity (for example HbA1c, blood pressure, cholesterol levels, albuminuria)<br>• Disease progression (age at diagnosis, duration of disease)<br>• Diabetes related complications (for example ocular bottom changes, kidney disease, neuropathy)<br>• Cardiovascular comorbidities<br>• Physical activity
**Undergoing surgical OA treatment**	diabetes	• Implant survival<br>• Reoperation within 2 years<br>• Change in patient reported outcome measures<br>• Adverse events (for example cardiovascular events)<br>• Mortality	• Patient characteristics (age, sex, BMI, smoking)<br>• Type of diabetes<br>• Diabetes medications<br>• Diabetes severity (for example HbA1c, blood pressure, cholesterol levels, albuminuria)<br>• Disease progression (age at diagnosis, duration of disease)<br>• Diabetes related complications (for example ocular bottom changes, kidney disease, neuropathy)<br>• Cardiovascular comorbidities
**Diabetes**			
	non-surgical OA treatment of hip and knee	• Diabetes Medications (diabetes, blood sugar, lipid and blood pressure lowering)<br>• Diabetes severity (for example HbA1, blood pressure, cholesterol levels, albuminuria)<br>• Diabetes related complications (for example ocular bottom changes, kidney disease, neuropathy)	• Patient characteristics (age, sex, BMI, smoking)<br>• OA severity (pain intensity, pain frequency, walking difficulties, quality of life, pain medications, sick leave)<br>• Cardiovascular comorbidities<br>• Physical activity
	surgical OA treatment of hip and knee	• Diabetes Medications (diabetes, blood sugar, lipid and blood pressure lowering)<br>• Diabetes severity (for example HbA1c, blood pressure, cholesterol levels, albuminuria)<br>• Diabetes related complications (for example ocular bottom changes, kidney disease, neuropathy)	• Patient characteristics (Age, Sex, Charnley classification, BMI)<br>• Surgical technique<br>• Implant characteristics<br>• Cardiovascular comorbidities

For peer review only

BMI: body mass index; OA: osteoarthritis; THR: total hip replacement; TKR: total knee replacement; HbA1: haemoglobin subunit alpha 1

432

For peer review only

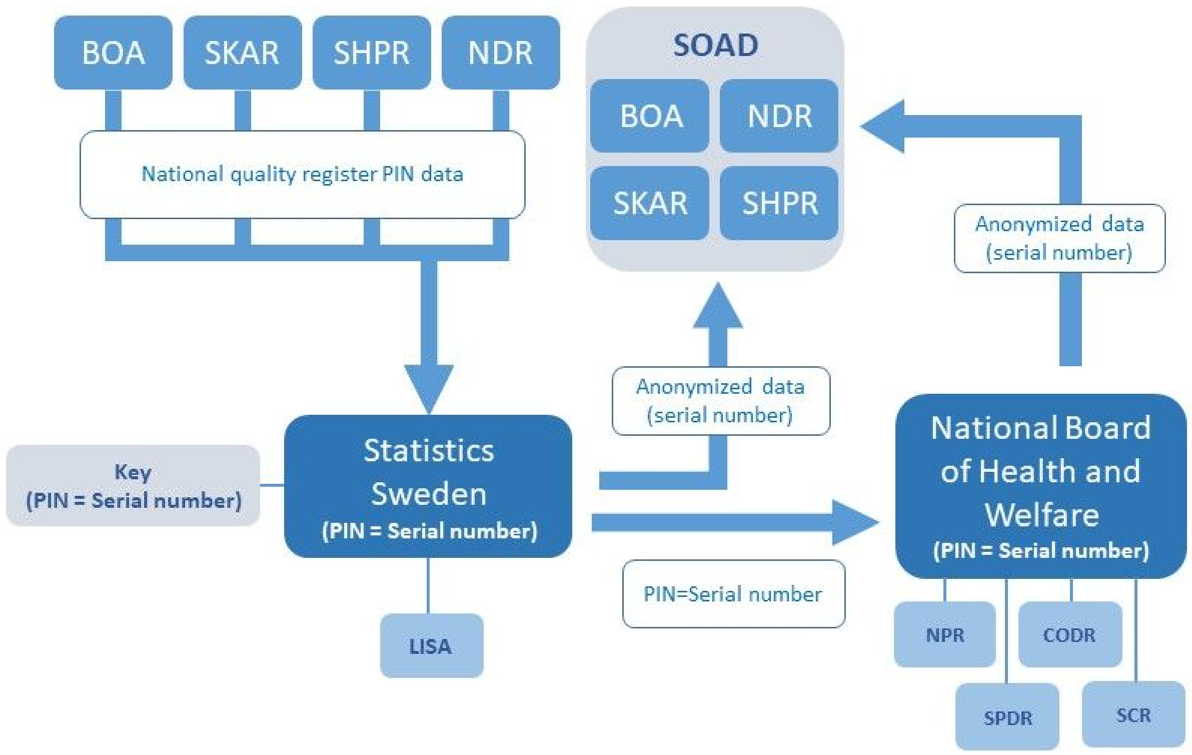

Figure 1. The data linkage process. Data from the four national quality registers Better Management of Patients with Osteoarthritis Register (BOA), Swedish Hip Arthroplasty Register (SHAR), Swedish Knee Arthroplasty Register (SKAR) and National Diabetes Register (NDR) is safely transferred to Statistics Sweden. Statistics Sweden will anonymise the data by replacing PIN with serial numbers. Data will be extracted from LISA (Longitudinal integration database for health insurance and labour market studies) and transferred to the entity principally responsible for the Swedish Osteoarthritis and Diabetes (SOAD) cohort research. The PIN and serial numbers will also be shared with National Board of Health and Welfare who will return data from National Patient Register (NPR), Swedish Prescribed Drug Register (SPDR), Cause of Death Register (CODR) and Swedish Cancer Register (SCR) to the entity principally responsible for the research. The linkage key will be saved at Statistics Sweden for 3 years to allow the possibility of adding more year cohorts or new variables to the research database if new research questions arise (with new ethical approval).

For peer review only

STROBE Statement—checklist of items that should be included in reports of observational studies

	Item No.	Recommendation	Page No.	Relevant text from manuscript
**Title and abstract**	1	(*a*) Indicate the study's design with a commonly used term in the title or the abstract	1-2	
		(*b*) Provide in the abstract an informative and balanced summary of what was done and what was found	2	
**Introduction**				
Background/rationale	2	Explain the scientific background and rationale for the investigation being reported	4-5	
Objectives	3	State specific objectives, including any prespecified hypotheses	5	
**Methods**				
Study design	4	Present key elements of study design early in the paper	6-8	
Setting	5	Describe the setting, locations, and relevant dates, including periods of recruitment, exposure, follow-up, and data collection	6-8	
Participants	6	(*a*) *Cohort study*—Give the eligibility criteria, and the sources and methods of selection of participants. Describe methods of follow-up<br>*Case-control study*—Give the eligibility criteria, and the sources and methods of case ascertainment and control selection. Give the rationale for the choice of cases and controls<br>*Cross-sectional study*—Give the eligibility criteria, and the sources and methods of selection of participants	7-8	
		(*b*) *Cohort study*—For matched studies, give matching criteria and number of exposed and unexposed<br>*Case-control study*—For matched studies, give matching criteria and the number of controls per case		
Variables	7	Clearly define all outcomes, exposures, predictors, potential confounders, and effect modifiers. Give diagnostic criteria, if applicable	9, table 5	
Data sources/ measurement	8*	For each variable of interest, give sources of data and details of methods of assessment (measurement). Describe comparability of assessment methods if there is more than one group	Table 1 to 4	
Bias	9	Describe any efforts to address potential sources of bias	9	
Study size	10	Explain how the study size was arrived at	Figure 1	

Continued on next page

For peer review only - http://bmjopen.bmj.com/site/about/guidelines.xhtml

Quantitative variables	11	Explain how quantitative variables were handled in the analyses. If applicable, describe which groupings were chosen and why	9	
Statistical methods	12	(*a*) Describe all statistical methods, including those used to control for confounding	9	
		(*b*) Describe any methods used to examine subgroups and interactions	9	
		(*c*) Explain how missing data were addressed	9	
		(*d*) *Cohort study*—If applicable, explain how loss to follow-up was addressed<br>*Case-control study*—If applicable, explain how matching of cases and controls was addressed<br>*Cross-sectional study*—If applicable, describe analytical methods taking account of sampling strategy	9	
		(*e*) Describe any sensitivity analyses		
**Results**				
Participants	13*	(a) Report numbers of individuals at each stage of study—eg numbers potentially eligible, examined for eligibility, confirmed eligible, included in the study, completing follow-up, and analysed		N/A
		(b) Give reasons for non-participation at each stage		N/A
		(c) Consider use of a flow diagram		N/A
Descriptive data	14*	(a) Give characteristics of study participants (eg demographic, clinical, social) and information on exposures and potential confounders		N/A
		(b) Indicate number of participants with missing data for each variable of interest		N/A
		(c) *Cohort study*—Summarise follow-up time (eg, average and total amount)		N/A
Outcome data	15*	*Cohort study*—Report numbers of outcome events or summary measures over time		N/A
		Case-control study—Report numbers in each exposure category, or summary measures of exposure		N/A
		Cross-sectional study—Report numbers of outcome events or summary measures		N/A
Main results	16	(*a*) Give unadjusted estimates and, if applicable, confounder-adjusted estimates and their precision (eg, 95% confidence interval). Make clear which confounders were adjusted for and why they were included		N/A
		(*b*) Report category boundaries when continuous variables were categorized		N/A
		(*c*) If relevant, consider translating estimates of relative risk into absolute risk for a meaningful time period		N/A

Continued on next page

For peer review only - http://bmjopen.bmj.com/site/about/guidelines.xhtml

For peer review only

Other analyses	17	Report other analyses done—eg analyses of subgroups and interactions, and sensitivity analyses	N/A
**Discussion**			
Key results	18	Summarise key results with reference to study objectives	N/A
Limitations	19	Discuss limitations of the study, taking into account sources of potential bias or imprecision. Discuss both direction and magnitude of any potential bias	N/A
Interpretation	20	Give a cautious overall interpretation of results considering objectives, limitations, multiplicity of analyses, results from similar studies, and other relevant evidence	N/A
Generalisability	21	Discuss the generalisability (external validity) of the study results	N/A
**Other information**			
Funding	22	Give the source of funding and the role of the funders for the present study and, if applicable, for the original study on which the present article is based	14

*Give information separately for cases and controls in case-control studies and, if applicable, for exposed and unexposed groups in cohort and cross-sectional studies.

Note: An Explanation and Elaboration article discusses each checklist item and gives methodological background and published examples of transparent reporting. The STROBE checklist is best used in conjunction with this article (freely available on the Web sites of PLoS Medicine at http://www.plosmedicine.org/, Annals of Internal Medicine at http://www.annals.org/, and Epidemiology at http://www.epidem.com/). Information on the STROBE Initiative is available at www.strobe-statement.org.

For peer review only

BMJ Open

Understanding the role of diabetes in the osteoarthritis disease and treatment process: a study protocol for The Swedish Osteoarthritis and Diabetes (SOAD) cohort

Journal:	*BMJ Open*
Manuscript ID	bmjopen-2019-032923.R1
Article Type:	Protocol
Date Submitted by the Author:	06-Nov-2019
Complete List of Authors:	Dell'Isola, Andrea; Lunds University Faculty of Medicine, Department of Clinical Sciences<br>Vinblad, Johanna; The Swedish Hip Arthroplasty Register, Centre of Registers Västra Götaland; Institute of Clinical Sciences, Department of Orthopaedics, Sahlgrenska Academy, University of Gothenburg<br>Lohmander, Stefan; Lunds University Faculty of Medicine, Department of Clinical Sciences<br>Svensson, Ann-Marie; Centre of Registers in Region Västra Götaland, National Diabetes Register; University of Gothenburg, Department of Molecular and Clinical Medicine<br>Turkiewicz, Aleksandra; Lund University, Dept of Orthopedics<br>Franzén, Stefan; Centre of Registers Västra Götaland, National Diabetes Register; University of Gothenburg, Health Metrics Unit, Sahlgrenska Academy<br>Nauclér, Emma; The Swedish Hip Arthroplasty Register, Centre of Registers Västra Götaland<br>W-Dahl, A; Lund University, Department of Clinical Sciences ; The Swedish Knee Arthroplasty Register<br>Abbott, Allan; Linkoping University, Department of Medical and Health Sciences (IMH)<br>Dahlberg, L; Lunds University Faculty of Medicine, Department of Clinical Sciences<br>Rolfson , Ola ; Institute of Clinical Sciences, Department of Orthopaedics, Sahlgrenska Academy, University of Gothenburg; The Swedish Hip Arthroplasty Register, Centre of Registers Västra Götaland<br>Englund, Martin; Lund University, Dept of Orthopedics
**Primary Subject Heading**:	Rheumatology
Secondary Subject Heading:	Epidemiology, Rehabilitation medicine
Keywords:	osteoarthritis, General diabetes < DIABETES & ENDOCRINOLOGY, register, cohort, exercise, SURGERY

SCHOLARONE™
Manuscripts

For peer review only - http://bmjopen.bmj.com/site/about/guidelines.xhtml

Understanding the role of diabetes in the osteoarthritis disease and treatment process: a study protocol for The Swedish Osteoarthritis and Diabetes (SOAD) cohort

Dell'Isola A[1]*, Vinblad J*[2,3], Lohmander LS[1], Svensson AM[5,6], Turkiewicz A[4], Franzén S[5,7], Nauclér E[2], W-Dahl A[1,8], Abbott A[9], Dahlberg L[1], Rolfson O[#2,3], Englund M[#,4].

* Shared first authors

# Shared senior authors

Corresponding author

Andrea Dell´Isola, Lund University, Faculty of Medicine, Department of Clinical Sciences, Orthopaedics, Lund, Sweden.
Email; andrea.dellisola@med.lu.se

1. Lund University, Faculty of Medicine, Department of Clinical Sciences Lund, Orthopaedics, Lund, Sweden.

2. The Swedish Hip Arthroplasty Register, Centre of Registers Västra Götaland, Sweden.

3. Department of Orthopedics, Institute of Clinical Science, Sahlgrenska Academy, University of Gothenburg, Sweden.

4. Lund University, Faculty of Medicine, Department of Clinical Sciences Lund, Orthopaedics, Clinical Epidemiology Unit, Lund, Sweden.

5. National Diabetes Register, Center of Registers, Gothenburg, Sweden

6. Department of Molecular and Clinical Medicine, University of Gothenburg, Gothenburg, Sweden

7. Health Metrics Unit, Sahlgrenska Academy, University of Gothenburg, Gothenburg, Sweden

8. The Swedish Knee Arthroplasty Register, Lund, Sweden

9. Department of Health, Medical and Caring Sciences, Division of Physiotherapy, Faculty of Medicine and Health Sciences, Linköping University, Linköping, Sweden

Keywords: Osteoarthritis, diabetes, register, cohort, exercise, surgery.

Word count: 3255

For peer review only - http://bmjopen.bmj.com/site/about/guidelines.xhtml

ABSTRACT (280 words)

Introduction

Osteoarthritis (OA) is the most common form of arthritis and a leading cause of disability worldwide. Metabolic comorbidities such as type 2 diabetes occur with a higher rate in people with OA than in the general population. Several factors including obesity, hyperglycemia toxicity and physical inactivity have been suggested as potential links between diabetes and OA, and have been shown to negatively impact patients' health and quality of life. However, little is known on the role of diabetes in determining the outcome of non-surgical and surgical management of OA, and at the same time, how different OA interventions may affect diabetes control. Thus, the overall aim of this project is to explore (1) the impact of diabetes on the outcome of non-surgical and surgical OA treatments, and (2) the impact of non-surgical and surgical OA treatments on diabetes control.

Methods and analysis

The study cohort is based on prospectively ascertained register data on a national level in Sweden. Data from OA patients who received a first-line non-surgical intervention and are registered in the National Quality Register for Better Management of Patients with Osteoarthritis will be merged with data from the Swedish Knee and Hip Arthroplasty Registers and the National Diabetes Register. Additional variables regarding patients' use of prescribed drugs, comorbidities, socioeconomic status, and cause of death will be obtained through other national health and population data registers. The linkage will be performed on an individual level using unique personal identity numbers.

Ethics and dissemination

This study received ethical approval (2019-02570) from the Swedish Ethical Review Authority. Results from this cohort will be submitted to peer-reviewed scientific journals and reported at the leading national and international meetings in the field.

For peer review only - http://bmjopen.bmj.com/site/about/guidelines.xhtml

ARTICLE SUMMARY

Strengths and limitations of this study

- This study will use a large nationwide population-based cohort based on data from national quality registers with high coverage and completeness to explore the relationship between diabetes and osteoarthritis (OA) and their related care process.
- We will include data regarding both non-surgical and surgical treatments for patients with OA giving the possibility to capture the influence of diabetes across the whole spectrum of OA treatments.
- We will include covariate information from several national registers that will allow to account for potential confounders and effect modifiers.
- A limitation of register-based studies is that the variables available and the characteristics of the treatments provided are predetermined, i.e., it is not possible to add covariates, exposures or outcomes (not in the registers) or to modify the interventions that have been given.
- People included in the National Quality Register for Better Management of Patients with Osteoarthritis (BOA) Register, the Swedish Hip Arthroplasty Register (SHAR), the Swedish Knee Arthroplasty Register (SKAR) received an intervention due to OA. Due to the complexity of the OA disease, treatments are individualised based on patient's and disease characteristics, which implies that selection bias and confounding by indication may bias our estimates.

For peer review only - http://bmjopen.bmj.com/site/about/guidelines.xhtml

INTRODUCTION

Osteoarthritis (OA) is the most common form of arthritis and affect mainly the knee and the hip joint [1]. In Sweden, more than 25% of the population aged >45 years is estimated to suffer from OA related pain symptoms and associated physical activity restrictions [2]. The average annual cost for a person affected by OA is reported to exceed €2,000, while the total European expense directly attributable to OA is estimated to be as high as €700 billion [3].

In addition to the already huge health and societal burden of OA, recent studies suggest that OA patients are twice as likely to have comorbidities compared to controls of the same age, indicating that the co-occurrence of multiple conditions in OA patients is the norm rather than the exception [4]. For instance, based on data showing a higher incidence of knee OA in overweight patients with metabolic disorders, a metabolic OA phenotype has been hypothesised [5-7]. Among the metabolic disorders, diabetes seems to play a central role due to its high prevalence and the toxic effect that hyperglycaemia has on the cartilage and its cells, and to the motor and sensory system through peripheral neuropathy [8-10].

According to data from the Swedish National Diabetes Register (NDR), approximately 5% of the Swedish population has diabetes, with type 2 diabetes accounting for approximately 90% of the cases [11]. Persons with diabetes have a higher risk of developing cardiovascular diseases and have a 2- to 5-fold increased risk of mortality compared with the general population [12].

In OA patients the prevalence of diabetes has been reported to be nearly three times higher than in the general population [7]. Obesity is a shared risk factor for OA of the knee and the hip and diabetes and can partially explain the association between these diseases [13, 14]. In addition to the mechanical overload caused by the excess weight, adipocytes release cytokines into the bloodstream promoting chronic low-grade inflammation and activating proteolytic enzymes which can trigger matrix degradation and initiate OA. At the same time, adipose-induced low-grade inflammation influences the metabolic dysregulation underlying several metabolic disorders among others, diabetes type II [14].

Once diabetes is initiated, it further promotes cartilage degeneration and joint inflammation causing enrichment of advanced glycation endproducts (AGEs) and matrix stiffening preventing optimal cushioning of the joint [7, 15]. This process leads to a worsening in OA symptoms promoting physical inactivity and weight gain and creating a vicious cycle which maintains the metabolic dysregulation and increases joint symptoms [13, 16-18] .

For peer review only - http://bmjopen.bmj.com/site/about/guidelines.xhtml

The evidence-based first-line management for people with hip and knee OA includes education and exercise, which are recommended regardless of OA disease severity, and weight loss for those overweight [19]. Metabolic comorbidities may have a significant impact on the treatment, partially explaining the lack of response experienced by some patients.

Replacement of the knee and of the hip is an effective treatment for patients with severe OA who do not sufficiently improve after non-surgical management [20]. Due to the rising prevalence of OA and the growing demand for this procedure, the number of hip and knee replacements has dramatically increased. In Sweden (total population 10 million), 14,700 primary total hip replacements (THRs) and nearly 14,000 total knee replacements (TKRs) were performed in 2017 with OA as indication. These figures account for 81% and 97% of the annual hip and knee replacements respectively, and translates in an annual incidence of nearly 150 procedures per 100,000 persons for both THR and TKR [21, 22].

Considering the association between diabetes and OA, surprisingly little is known regarding the influence that diabetes has on the outcome of OA treatments (both non-surgical and surgical) [23, 24]. In addition, no evidence exists regarding the effect that OA treatments (both non-surgical and surgical) may have on diabetes control (both for type I and type II). Thus, merging data from multiple Swedish registers will allow us to follow patients with knee and hip OA through the progress of their disease to understand how diabetes influences the OA disease process. This study cohort is created to increase knowledge of the influence that diabetes has on the outcomes of OA patients who have received non-surgical and/or surgical treatments for hip and knee OA, and the influence that hip and knee OA and its treatments have on the diabetes control.

METHODS AND ANALYSIS

Research questions

In order to understand how the coexistence of OA of the hip or of the knee and diabetes influences the treatment effects in these diseases, a series of research questions have been posed. The research questions cover two main thematic areas: (1) the impact of diabetes on the outcome of non-surgical and surgical OA treatments, and (2) the impact of OA non-surgical and surgical OA treatments on diabetes control (consideration to type of diabetes (I or II) will be taken).

<u>Area 1</u>

1. What is the prevalence of diabetes in people with OA undergoing a non-surgical intervention?

For peer review only - http://bmjopen.bmj.com/site/about/guidelines.xhtml

2. Is the presence of diabetes, diabetes-related factors (e.g. type of diabetes, diabetes-related medication, blood pressure, HbA1c) associated with OA severity (e.g. pain intensity, pain frequency, walking difficulties) of people with OA undergoing a self-management non-surgical intervention?
3. Is the presence of diabetes and diabetes-related factors associated with the outcomes of a self-management non-surgical intervention for people with OA (e.g. change in pain levels, pain frequency, walking difficulties)?
4. Is the presence of diabetes and diabetes-related factors associated with the risk of joint replacement in people with OA who underwent a self-management non-surgical intervention?
5. What is the incidence of reoperations and other adverse events such as thromboembolism, cardiovascular events and mortality following primary total hip or knee replacement due to OA in people with or without diabetes?
6. What diabetes-related factors are associated with the risk of reoperation and other adverse events following primary total hip or knee replacement among person with diabetes?

<u>Area 2</u>

7. How does a self-management non-surgical intervention for OA influence diabetes (type I vs type II) control (e.g. change in diabetes drug intake after the intervention, change in Hb A1c levels after the intervention) compared to comparable people with diabetes who had not taken part in the intervention?
8. How does primary total hip or knee replacement influence the diabetes control compared to comparable persons with diabetes but with no history of hip or knee arthroplasty?
9. What diabetes-related risk factors are associated with diabetes control following primary total hip or knee replacement due to OA?

For peer review only

For peer review only - http://bmjopen.bmj.com/site/about/guidelines.xhtml

Main exposures and outcomes

The exposure and outcome measures are described in table 1. Potential confounding factors for main analysis and disease sub analysis are described with examples.

INSERT TABLE 1 HERE

The Swedish OA and Diabetes cohort (SOAD)

This nationwide observational study cohort (SOAD) will be based on prospectively obtained individual-level data from four main sources: the National Quality Register for Better Management of Patients with Osteoarthritis (BOA) Register, the Swedish Hip Arthroplasty Register (SHAR), the Swedish Knee Arthroplasty Register (SKAR), and the Swedish National Diabetes Register (NDR). Data starting from the year of each register establishment will be merged using the unique personal identity number (PIN) issued to all legal residents in Sweden. Additional variables regarding patients' use of prescribed drugs, comorbidities, cause of death, and socioeconomic information will be obtained through the following population-based registers:

- The Swedish Prescribed Drug Register (SPDR) held by the National Board of Health and Welfare.
- The National Patient Register (NPR) held by the National Board of Health and Welfare; information regarding in-hospital diagnoses and outpatient specialist care diagnoses, e.g. interventions, adverse events such as thromboembolism or other comorbid conditions.
- Swedish Cancer Register, The National Board of Health and Welfare.
- The Cause of death Register (CODR) held by the National Board of Health and Welfare
- Longitudinal integration database for health insurance and labour market studies (LISA) held by Statistics Sweden for data such as marital status, educational level and country of origin.

Data sources

BOA: The BOA register was started in 2008 and currently includes more than 100,000 individuals with OA who have registered for an evidence-based self-management programme. These patients sought treatment for knee and/or hip pain in primary healthcare in Sweden and were referred for standardised core treatment (education and supervised exercises) after a confirmed clinical/radiographic OA diagnosis in accordance with the recommendations for OA diagnosis from the Swedish National Board of Health and Welfare [25]. These guidelines are in line with internationally accepted diagnostic criteria, and according to the guidelines, radiographic examination should only be used in uncertain cases, if the

For peer review only

patient is not responding to treatment or when a surgical intervention is planned [26, 27]. BOA offers all the patients two education sessions focusing on the pathophysiology of OA and the benefit of exercise which are mandatory for participating in the second (exercise) of the programme. A third, optional, session held by a trained osteoarthritis communicator (a patient with osteoarthritis who previously participated in BOA) is offered to provide a patient's perspective on OA self-management and to teach about the lived experience with this condition, as well as his or her personal experience of non-surgical interventions. After the education, participants can take part in the exercise phase of BOA which consists of a face-to-face session with a physiotherapist. In this session, the patients receive a personalised intervention programme and the necessary instructions to perform it independently at home. Thereafter, participants are given the possibility to perform their exercise programme on their own or to participate in up to 12 supervised group exercise session with a physiotherapist provided two times a week for six weeks. Thus, the register contains two separate cohorts that performed, in addition to the education sessions, either home exercise or supervised exercise. The register has a data completeness of almost 90% and the BOA participants have answered validated and patient-relevant socio-demographic and outcome questionnaires at baseline, after the interventions (two to five months) and at one year (12-15 months) (Table 2).

INSERT TABLE 2 HERE

SHAR: Started in 1979, SHAR registers primary hip replacement operations and reoperations in Sweden including individual patient data, surgical technique and type of implant used. Since 2002 patient-reported measures such as joint pain, HRQoL and satisfaction with treatment have also been collected before surgery and one, six and ten years postoperatively. The register encompasses 318 000 primary total hip replacements due to OA and 61 500 reoperations after total hip replacements where OA was the main reason for the primary surgery (at the end of 2018). The register has overall data completeness of 98,5% (2016) including all indications for total hip replacements (Table 3).

SKAR: The Swedish Knee Arthroplasty Register is a Swedish National Quality Register founded in 1975. The register collects individual patient data, surgical technique and type of implant used for patients who undergo knee replacement. The SKAR also collects information on re-operations/revision surgery. SKAR has completeness of 98.1 % (2016) and have registered almost 270,000 primary knee replacements due to OA and more than 21 400 revisions at the end of 2018 (Table 4).

INSERT TABLE 3 AND 4 HERE

For peer review only - http://bmjopen.bmj.com/site/about/guidelines.xhtml

<u>NDR:</u> NDR has been a Swedish National Quality Register since 1996 and collects data on clinical characteristics, risk factors, laboratory analyses, complications of diabetes, and medications for patients 18 years of age or older with a diagnosis of diabetes (Table 5). The completeness is 96.5 % (2017) and the register has 750 004 (2017) unique individuals in their database. More than 95% of all individuals with type 1 diabetes mellitus (T1DM) and 90% of individuals with type 2 diabetes mellitus (T2DM) in Sweden are included in the NDR.

INSERT TABLE 5 HERE

Data linkage

<u>Personal Identity Number (PIN):</u> In Sweden, all legal residents are registered with a unique PIN that provides information on the date of birth and sex. Swedish law requires all documentation regarding healthcare contacts to be registered using the patient's PIN [28]. The PIN is also used for registration of data for statistics such as national population-based registers and healthcare quality registers [29, 30]. The system allows for linkage of data at an individual level between the different registers in Sweden with the possibility of creating merged research databases for epidemiological research on large populations, after the relevant ethical approval has been obtained. Data linkage for the current study will include all the data available in BOA, SHPR, SKAR and NDR and it will start from the first time point available in the registers. The data linkage process has been initiated and it is described in figure 1. Data linkage is expected to be completed by 2020. Estimated start and end dates for the project are 01/09/2020 and 01/09/2030 respectively.

INSERT HERE FIGURE 1

Analysis plan

Data harmonisation will be performed, the more reliable data source will be used to guarantee information quality and reliability across exposed and unexposed subjects. To establish the reliability of the source we will consider how the measurement was performed (e.g self-reported vs measured) and data quality (e.g. percentage of missing). If the same variable (e.g. BMI) will be present in the source deemed as most reliable at more than one time-point, we will use the measurement closest to the time point of interest.

We will develop a specific statistical analysis plan for each specific study that will be conducted within SOAD. These will follow several general principles. We will aim for the inclusion of all available knee and

For peer review only - http://bmjopen.bmj.com/site/about/guidelines.xhtml

hip OA and diabetes patients to limit potential selection bias. We will use multiple imputation methods to impute the missing data on exposures, outcomes and confounders, when relevant. The imputation model will be specific for each study and compatible with the chosen analysis model. For example, the fully conditional specification (also called chained equations) may be used to enable flexible models for proper imputation of all variables. In the statistical modelling we will aim for estimation of causal effects and statistical models will be chosen accordingly using direct acyclic graphs (DAGs) to enable proper confounding control [31]. For confounding control, we will use regression models or inverse probability weighting. For analysis of panel data (i.e. longitudinal repeated measurements of the participants and/or data clustered by caregiver) we will use multilevel regression models. For time-to-event data we will use the proportional hazards Cox regression model, or, if appropriate, parametric models. For mediation analysis, we will use linear models or maximum likelihood structural equation models when appropriate. For categorical outcomes we will use other approaches [32, 33]. We will report the results from all analyses as relevant estimated effect size (such as risk differences, risk ratios of hazard ratios) with 95% confidence intervals and interpret these for clinical relevance, irrespectively of statistical significance [34, 35].

For the current study, we did not perform any power calculation. This because the question of power can be considered secondary in such a setting where the sample size is driven by data availability and not decided a priori. In addition, when interpreting results, we will not use the concept of statistical significance, but we will base our interpretation on effect sizes (and the uncertainty around them) and clinical relevance [36]. Therefore, we will include all the available data, with ~100,000 BOA participants, whereof an estimated 15,000 have diabetes [37]. Regarding the SHAR and SKAR, we will have data for 240 000 and 320 000 joint replacements respectively. Based on previous studies we expect that the prevalence of diabetes will be around 8% and 14% among patients undergoing THR and TKR, respectively [38, 39]. We expect that this will enable precise estimations of the main effects of interest.

Patient and public involvement

Patient representatives were not involved in the development of the research question or the design of this study. However, patients were involved in the creation of the BOA supported self-management programme and contributed to the development of the key contents of the programme [40]. Patients are also actively involved in the BOA programme and deliver a compulsory education session where the patient’s perspective on OA self-management treatment is explored. The Swedish Hip and Knee Arthroplasty Registers have patient representatives on their respective steering committees.

For peer review only - http://bmjopen.bmj.com/site/about/guidelines.xhtml

ETHICS AND DISSEMINATION

Storage and management of data

Ethical approval for the creation of the cohort and the analyses as detailed in the present protocol has been obtained (14th May 2019, 2019-02570). A copy of the full data set will be stored at the Center of Registers Västra Götaland, Gothenburg, Sweden. A second copy of the full data set will be stored at Lund University on the platform LUSEC (Lund information security platform). The platforms are designed to securely store, manage and analyse data in accordance with the European Union general data protection regulation (GDPR). The process of linkage, storage and management of data, the role of informed consent in register-based research and safeguarding the integrity of study participants follows the legal and ethical frameworks as described by Swedish law and ethical boards. This has been described by Ludvigsson et al [28].

Dissemination

The results from this study will be published in peer-reviewed scientific journals and will be presented at the leading national and international meetings in the field. The results will also be disseminated through annual reports published on the registers' websites in order to reach clinicians working with people with OA and diabetes.

In order to reach people suffering from OA and diabetes, we aim to exploit the connection between BOA people seeking care for OA. Briefly, we will target BOA educators providing them material (through email and mail) regarding the progress and achievement of the project focusing on the impact that the coexistence of OA and diabetes has on the treatment. In this way, we will reach the new patients taking part in BOA that will be better informed about their condition and about the strategy to undertake in order to maximise the benefit of first-line intervention.

Finally, In SOAD we recognize the importance of reaching a broad audience, for this reason, we will utilise the authors Twitter and Facebook networks to create awareness among the general public of the importance of the issue. The social media will help us to make scientific practice easily accessible and understandable to an audience of non-specialists.

For peer review only - http://bmjopen.bmj.com/site/about/guidelines.xhtml

DISCUSSION

This study cohort will provide unique insights into the relationships between diabetes and OA. By using data from the BOA, SHAR, SKAR and NDR registers we will be able to investigate the influence of diabetes on the outcome of non-surgical and surgical OA interventions as well as the effect of OA treatments on diabetes control.

To our knowledge, this will be the largest dataset combining data on OA and diabetes management. Thanks to the large sample size on a national level, results arising from this study will likely have high external validity and generalisability. However, treatment data collected in national registers are likely to be influenced by the regional differences in treatment protocols and data collection that characterise different clinical environments when compared to e.g. highly standardised clinical trials.

In conclusion, to optimise treatments for OA and diabetes and move towards a personalised-care approach, it is important to identify factors and comorbidities that may negatively influence the outcome of the interventions. The coexistence of several conditions creates a more complex disease status which requires additional considerations and cares for the patient to experience the desired benefit from the provided interventions. The SOAD cohort will help us to identify these patients with complex needs, opening a venue for the development of better treatment approaches. Ultimately, the cohort has the potential to impact on the way OA is managed when other comorbidities coexist, potentially reducing the huge burden of this disease.

For peer review only

For peer review only - http://bmjopen.bmj.com/site/about/guidelines.xhtml

References

1. European Medical Agency (EMA); Guideline on clinical investigation of medicinal products used in the treatment of osteoarthritis. https://www.ema.europa.eu; Accessed Sep 24 2019 2010.
2. Turkiewicz A, Petersson IF, Bjork J, Hawker G, Dahlberg LE, Lohmander LS, et al. Current and future impact of osteoarthritis on health care: a population-based study with projections to year 2032. Osteoarthritis Cartilage 2014; 22: 1826-1832.
3. Salmon JH, Rat AC, Sellam J, Michel M, Eschard JP, Guillemin F, et al. Economic impact of lower-limb osteoarthritis worldwide: a systematic review of cost-of-illness studies. Osteoarthritis Cartilage 2016; 24: 1500-1508.
4. Kadam UT, Jordan K, Croft PR. Clinical comorbidity in patients with osteoarthritis: A case-control study of general practice consumers in England and Wales. Annals of the Rheumatic Diseases 2004; 63: 408-414.
5. Dell'Isola A, Allan R, Smith SL, Marreiros SS, Steultjens M. Identification of clinical phenotypes in knee osteoarthritis: a systematic review of the literature. BMC Musculoskelet Disord 2016; 17: 425.
6. Dell'Isola A, Steultjens M. Classification of patients with knee osteoarthritis in clinical phenotypes: Data from the osteoarthritis initiative. PLoS One 2018; 13: e0191045.
7. Berenbaum F. Diabetes-induced osteoarthritis: from a new paradigm to a new phenotype. Annals of the Rheumatic Diseases 2011; 70: 1354-1356.
8. Zhuo Q, Yang W, Chen J, Wang Y. Metabolic syndrome meets osteoarthritis. Nat Rev Rheumatol 2012; 8: 729-737.
9. Yoshimura N, Muraki S, Oka H, Kawaguchi H, Nakamura K, Akune T. Association of knee osteoarthritis with the accumulation of metabolic risk factors such as overweight, hypertension, dyslipidemia, and impaired glucose tolerance in Japanese men and women: the ROAD study. J Rheumatol 2011; 38: 921-930.
10. Karvonen-Gutierrez CA, Sowers MR, Heeringa SG. Sex dimorphism in the association of cardiometabolic characteristics and osteophytes-defined radiographic knee osteoarthritis among obese and non-obese adults: NHANES III. Osteoarthritis Cartilage 2012; 20: 614-621.
11. Gudbjörnsdottier S SA, Eliasson B, Eeg Olofsson K, Linder E, Samuelsson P, Miftaraj M. Nationella Diabetesregistrets årsrapport. 2017.
12. Thomas RJ, Palumbo PJ, Melton III LJ, Roger VL, Ransom J, O'brien PC, et al. Trends in the mortality burden associated with diabetes mellitus: a population-based study in Rochester, Minn, 1970-1994. Archives of Internal Medicine 2003; 163: 445-451.
13. Wluka AE, Lombard CB, Cicuttini FM. Tackling obesity in knee osteoarthritis. Nat Rev Rheumatol 2013; 9: 225-235.
14. Hotamisligil GS. Inflammation, metaflammation and immunometabolic disorders. Nature 2017; 542: 177-185.
15. Rosa SC, Goncalves J, Judas F, Mobasheri A, Lopes C, Mendes AF. Impaired glucose transporter-1 degradation and increased glucose transport and oxidative stress in response to high glucose in chondrocytes from osteoarthritic versus normal human cartilage. Arthritis Res Ther 2009; 11: R80.
16. Mobasheri A. Glucose: an energy currency and structural precursor in articular cartilage and bone with emerging roles as an extracellular signaling molecule and metabolic regulator. Front Endocrinol (Lausanne) 2012; 3: 153.
17. Berenbaum F, Wallace IJ, Lieberman DE, Felson DT. Modern-day environmental factors in the pathogenesis of osteoarthritis. Nature Reviews Rheumatology 2018.

For peer review only - http://bmjopen.bmj.com/site/about/guidelines.xhtml

18. Verzijl N, Bank RA, TeKoppele JM, DeGroot J. AGEing and osteoarthritis: a different perspective. Curr Opin Rheumatol 2003; 15: 616-622.
19. Fernandes L, Hagen KB, Bijlsma JW, Andreassen O, Christensen P, Conaghan PG, et al. EULAR recommendations for the non-pharmacological core management of hip and knee osteoarthritis. Ann Rheum Dis 2013; 72: 1125-1135.
20. Skou ST, Roos EM, Laursen MB, Rathleff MS, Arendt-Nielsen L, Rasmussen S, et al. Total knee replacement and non-surgical treatment of knee osteoarthritis: 2-year outcome from two parallel randomized controlled trials. Osteoarthritis Cartilage 2018; 26: 1170-1180.
21. Kärrholm JMM. OD, Vinblad J, Rogmark C, Rolfson O. Svenska Höftprotesregistret Årsrapport 2017. https://shpr.demo.registercentrum.se/ 2018.
22. Robertsson O W-DA, Lidgren L, Sundberg M. Årsrapport 2018 Svenska Knäprotesregistret http://www.myknee.se/ 2018.
23. Malinzak RA, Ritter MA, Berend ME, Meding JB, Olberding EM, Davis KE. Morbidly obese, diabetic, younger, and unilateral joint arthroplasty patients have elevated total joint arthroplasty infection rates. J Arthroplasty 2009; 24: 84-88.
24. Robertson F, Geddes J, Ridley D, McLeod G, Cheng KJTK. Patients with Type 2 diabetes mellitus have a worse functional outcome post knee arthroplasty: a matched cohort study. 2012; 19: 286-289.
25. Socialstyrelsen. Nationella riktlinjer för rörelseorganens sjukdomar 2012: Osteoporos, artros, inflammatoriskryggsjukdom och ankyloserande spondylit, psoriasisartrit och reumatoid artrit: Stöd för styrning och ledning. Socialstyrelsen 2012.
26. Altman R, Alarcon G, Appelrouth D, Bloch D, Borenstein D, Brandt K, et al. The American College of Rheumatology criteria for the classification and reporting of osteoarthritis of the hip. 1991; 34: 505-514.
27. Altman R, Asch E, Bloch D, Bole G, Borenstein D, Brandt K, et al. Development of criteria for the classification and reporting of osteoarthritis: Classification of osteoarthritis of the knee. Arthritis & Rheumatism 1986; 29: 1039-1049.
28. Ludvigsson JF, Otterblad-Olausson P, Pettersson BU, Ekbom A. The Swedish personal identity number: possibilities and pitfalls in healthcare and medical research. Eur J Epidemiol 2009; 24: 659-667.
29. Ludvigsson JF, Almqvist C, Bonamy AK, Ljung R, Michaelsson K, Neovius M, et al. Registers of the Swedish total population and their use in medical research. Eur J Epidemiol 2016; 31: 125-136.
30. Ludvigsson JF, Andersson E, Ekbom A, Feychting M, Kim JL, Reuterwall C, et al. External review and validation of the Swedish national inpatient register. BMC Public Health 2011; 11: 450.
31. Textor J, van der Zander B, Gilthorpe MS, Liskiewicz M, Ellison GT. Robust causal inference using directed acyclic graphs: the R package 'dagitty'. Int J Epidemiol 2016; 45: 1887-1894.
32. Breen R, Karlson KB, Holm A. Total, direct, and indirect effects in logit and probit models. Sociological Methods & Research 2013; 42: 164-191.
33. Lange T, Hansen KW, Sorensen R, Galatius S. Applied mediation analyses: a review and tutorial. Epidemiol Health 2017; 39: e2017035.
34. Amrhein V, Greenland S, McShane B. Scientists rise up against statistical significance. Nature Publishing Group 2019.
35. McShane BB, Gal D, Gelman A, Robert C, Tackett JL. Abandon Statistical Significance. The American Statistician 2019; 73: 235-245.
36. Wasserstein RL, Schirm AL, Lazar NA. Moving to a World Beyond "p < 0.05". The American Statistician 2019; 73: 1-19.

For peer review only - http://bmjopen.bmj.com/site/about/guidelines.xhtml

37. Louati K, Vidal C, Berenbaum F, Sellam J. Association between diabetes mellitus and osteoarthritis: systematic literature review and meta-analysis. RMD open 2015; 1: e000077-e000077.
38. Pedersen AB, Mehnert F, Johnsen SP, Sorensen HT. Risk of revision of a total hip replacement in patients with diabetes mellitus: a population-based follow up study. J Bone Joint Surg Br 2010; 92: 929-934.
39. Lenguerrand E, Beswick AD, Whitehouse MR, Wylde V, Blom AW. Outcomes following hip and knee replacement in diabetic versus nondiabetic patients and well versus poorly controlled diabetic patients: a prospective cohort study. Acta Orthop 2018; 89: 399-405.
40. Thorstensson CA, Garellick G, Rystedt H, Dahlberg LE. Better Management of Patients with Osteoarthritis: Development and Nationwide Implementation of an Evidence-Based Supported Osteoarthritis Self-Management Programme. Musculoskeletal Care 2015; 13: 67-75.

Author contribution

DA, VJ, LLS, SAM, TA, FS, NE, WA, AA, DL, RO, EM provided substantial contributions to the conception, design of the work and analysis plan of data. DA, VJ, LLS, SAM, TA, FS, NE, WA, AA, DL, RO, EM contributed to the drafting of the protocol and approved the final version.

Funding

JV has received funding from Dr. Felix Neuberghs Foundation. ME and AT are funded by the Swedish Research Council and The Swedish Rheumatology Association.

Data sharing

All information regarding individual patients is subject to confidentiality in accordance with The Public Access to Information and Secrecy Act (SFS 2009:400) chapter 25 article 1. Therefore, we will be unable to share any data included in the SOAD cohort.

Conflict of interest

AWD is employed at the Swedish Knee Arthroplasty Register (SKAR). JV, AMS, SF, EN, OR are employed by the Centre of Registers Västra Götaland, Sweden. AWD is employed at the Swedish Knee Arthroplasty Register (SKAR). LD is the Co-founder and Chief Medical Officer of Joint Academy, a company which provides web-based non-surgical interventions for patients with hip and knee osteoarthritis. AA is employed by the Better management of OsteoArthritis register (BOA). All the other authors have nothing to disclose

For peer review only

For peer review only - http://bmjopen.bmj.com/site/about/guidelines.xhtml

Table 1. Exposure and outcome for the study populations and examples of confounders and effect modifiers for the study analyses.

Population	Exposure	Outcome	Example confounders and effect modifiers
**People with OA**			
**undergoing self-management treatment**	diabetes	• Pain intensity<br>• Pain frequency<br>• Walking difficulties<br>• Quality of life<br>• Use of pain medications<br>• Sick leave	• Patient characteristics (age, sex, BMI, smoking)<br>• Type of diabetes<br>• Diabetes medications<br>• Diabetes severity (for example HbA1c, blood pressure, cholesterol levels, albuminuria)<br>• Diabetes disease duration (age at diagnosis, duration of disease)<br>• Diabetes related complications (for example ocular bottom changes, kidney disease, neuropathy)<br>• Cardiovascular comorbidities<br>• Physical activity<br>• Weight Change
**Undergoing surgical OA treatment**	diabetes	• Implant survival<br>• Reoperation within 2 years<br>• Change in patient reported outcome measures<br>• Adverse events (for example cardiovascular events)<br>• Mortality	• Patient characteristics (age, sex, BMI, smoking)<br>• Type of diabetes<br>• Diabetes medications<br>• Diabetes severity (for example HbA1c, blood pressure, cholesterol levels, albuminuria)<br>• Disease progression (age at diagnosis, duration of disease)<br>• Diabetes related complications (for example ocular bottom changes, kidney disease, neuropathy)<br>• Cardiovascular comorbidities<br>• Weight Change
**Diabetes**			
	non-surgical OA treatment of hip and knee	• Diabetes Medications (diabetes, blood sugar, lipid and blood pressure lowering)<br>• Diabetes severity (for example HbA1, blood pressure, cholesterol levels, albuminuria)<br>• Diabetes related complications (for example ocular bottom changes, kidney disease, neuropathy)	• Patient characteristics (age, sex, BMI, smoking)<br>• OA severity (pain intensity, pain frequency, walking difficulties, quality of life, pain medications, sick leave)<br>• Type of diabetes<br>• Cardiovascular comorbidities<br>• Physical activity<br>• Weight Change
	surgical OA treatment of hip and knee	• Diabetes Medications (diabetes, blood sugar, lipid and blood pressure lowering)<br>• Diabetes severity (for example HbA1c, blood pressure, cholesterol levels, albuminuria)<br>• Diabetes related complications (for example	• Patient characteristics (Age, Sex, Charnley classification, BMI)<br>• Type of diabetes<br>• Surgical technique<br>• Implant characteristics<br>• Cardiovascular comorbidities<br>• Weight Change

For peer review only

For peer review only - http://bmjopen.bmj.com/site/about/guidelines.xhtml

ocular bottom changes,
kidney disease, neuropathy)

BMI: body mass index; OA: osteoarthritis; THR: total hip replacement; TKR: total knee replacement; HbA1: haemoglobin subunit alpha 1

442

For peer review only

For peer review only - http://bmjopen.bmj.com/site/about/guidelines.xhtml

Table 2. Description of the single variables collected from the Better Management of Patients with Osteoarthritis Register (BOA)

**BOA Register**		Baseline	Evaluation 3 months	Evaluation 12 months
**Variable category**	**Variable**			
Date	Date of visits	x	x	x
Patient-reported measures	Age, sex, weight, height	x		
	Smoking	x		
	Most affected joint (hip, knee or hand)	x	x	x
	Other affected joints	x	x	x
	Fear avoidance	x	x	x
	Request for surgery	x	x	x
	Walking difficulties	x	x	x
Physical activity level	Duration of physical training [a)]	x	x	x
	Duration of physical activity [b)]	x	x	x
Satisfaction	Satisfaction with treatment		x	x
Musculoskeletal comorbidity	Charnley class [c)]	x	x	x
Pain	Pain severity [d)] NRS	x	x	x
	Pain frequency	x	x	x
Generic	EQ-5D	x	x	x
Self-efficacy	Arthritis self-efficacy scale	x	x	x
Physiotherapist-reported measures	Earlier radiography/MRI/surgery in the most affected or the contralateral joint	x		
	Earlier treatments (including physiotherapy/adapted training/information on weight reduction/pharmaceuticals)	x		
	Waiting list for surgery	x	x	
	Use of medications for OA	x	x	
Follow-up	Radiography/MRI/surgery in the most affected or the contralateral joint since last evaluation		x	
	Compliance with intervention		x	

For peer review only - http://bmjopen.bmj.com/site/about/guidelines.xhtml

[a] Answering to the question:“ During a regular week, how much time do you spend exercising on a level that makes you short winded, for example running, fitness class, or ball games?” graded on categorical scale from “0” to “more than 120 minutes”.
[b] Answering to the question: “During a regular week, how much time are you physically active in ways that are not exercise, for example walks, bicycling, or gardening?” graded on categorical scale from “0” to “more than 300 minutes”.[25]
[c] Charnley class: classifications of musculoskeletal impairment. Class A corresponds with unilateral hip or knee OA; class B bilateral hip or knee OA and class C indicates multiple joint OA or some other condition that inhibits the patient’s ability to walk
[d] Answering to: "Select the box that corresponds to your average pain from your most affected joint the last week".
NRS: Numeric Rating Scale, VAS: visual analogue scale, MRI: magnetic resonance imaging

For peer review only

For peer review only - http://bmjopen.bmj.com/site/about/guidelines.xhtml

Table 3. Description of single variables collected from the Swedish Hip Arthroplasty Register

**Swedish Hip Arthroplasty Register**		**Baseline**	**Follow-up 1, 6 and 10 year**
**Variable category**	**Variables**		
Surgery-related variables			
Diagnosis (at hip)	ICD-10	x	
	Laterality	x	
Date	Date of surgery	x	
Type of surgery	Primary, revision, other reoperation	x	
Type of replacement	Total, partial, resurfacing hip replacement	x	
Patient status	Age, sex, height , weight, ASA class	x	
Implant characteristics	Article number, type of implant	x	
Technique	Incision, fixation	x	
Patient-reported measures			
Smoking status	Smoking (never, ex, daily, not daily))	x	
Musculoskeletal comorbidity	Charnley class [a)]	x	x
		x	x
Generic	EQ-5D	x	x
Treatment before hip replacement surgery	Physiotherapy	x	
	Standardised core treatment of education and supervised exercises	x	
Disease specific	Hip pain (Likert)	x	x
Satisfaction	Satisfaction with treatment (Likert)		x

[a)] Charnley class: classifications of musculoskeletal impairment. Class A corresponds to unilateral hip disease; Class B bilateral hip disease; and Class C indicates multiple joint disease or some other condition that inhibits the patient's ability to walk.
ICD-10, International Classification of Diseases, tenth revision; VAS, visual analogue scale. ASA-class, American Society of Anesthesiologists physical status classification system

For peer review only

Table 4. Description of single variables collected from the Swedish Knee Arthroplasty Register

**Swedish Knee Arthroplasty Register**		Baseline	Follow-up 1 year
**Variable category**	**Variable**		
Surgery-related variables			
Diagnosis (at knee)	ICD-10	x	
	Laterality	x	
Date	Date of surgery	x	
Type of surgery	Primary, revision	x	
Type of replacement	Total, uni-compartmental, stabilised (hinged) knee replacement	x	
Patient status	Age, sex, height, weight, ASA class	x	
Implant characteristics	Article number, type of implant	x	
Technique	Incision, fixation	x	
Patient-reported measures			
Musculoskeletal comorbidity	Charnley class (modified) [a)]	x	x
		x	x
Generic	EQ-5D	x	x
Satisfaction	Satisfaction with treatment (VAS)		x
Disease specific	Knee injury and Osteoarthritis Outcome Score (KOOS), Knee pain (VAS)	x	x

[a)] Charnley class: classifications of musculoskeletal impairment. Class A: unilateral knee disease; Class B1:bilateral OA, one knee is scheduled for or already received arthroplasty surgery while the other knee has OA but no scheduled arthroplasty surgery; B2: bilateral OA, one knee is scheduled for or already received arthroplasty surgery while the other knee has already received knee arthroplasty surgery; Class C to multiple joint disease or some other condition that inhibits the patient's ability to walk.
ICD-10, International Classification of Diseases, tenth revision; VAS, visual analogue scale. ASA-class, American Society of Anesthesiologists physical status classification system

For peer review only

For peer review only - http://bmjopen.bmj.com/site/about/guidelines.xhtml

Table 5. Description of single variables collected from the National Diabetes Register

National diabetes register

**Variable category**	**Variable**
Patient's characteristics	Age (years), sex, height, weight, BMI
Diabetes characteristics	Type of diabetes, HbA1c(mmol/mol), debut year of diabetes, diabetes duration (years), age at onset
Diabetes treatment	Diet only, insulin, tablets, tablets and insulin
Method of insulin delivery	Insulin Pump Treatment (CSI), multiple daily injections (MDI)
Blood pressure	Systolic blood pressure (mmHg), Diastolic blood pressure (mmHg)
Cholesterol	Total cholesterol (mmol/L), LDL (mmol/L), HDL (mmol/L)
HbA1c	Triglycerides (mmol/L)
Renal function	Creatinine (µmol/L), eGFR (mL/min/1.73 m2)
Retinopathy	Retinopathy (yes/no)
Other treatments	Anti-hypertensive treatment, Lipid-lowering treatment,
Physical Activity	Times per week of moderate to intense physical activity
Smoking status	Smoking (yes/no)
Albuminuria	Micro albuminurea, macro albuminurea (%)

Variables are measured at least once per year for patients with diabetes type 2 and four times per year for patients with diabetes type 1. If the patient has specific problems variables may be recorded with higher frequency. BMI: body mass index; HbA1: Haemoglobin subunit alpha 1; LDL: low density lipoprotein, HDL: high density lipoprotein; eGFR: estimated glomerular filtration rate

For peer review only

Figure 1. The data linkage process. Data from the four national quality registers Better Management of Patients with Osteoarthritis Register (BOA), Swedish Hip Arthroplasty Register (SHAR), Swedish Knee Arthroplasty Register (SKAR) and National Diabetes Register (NDR) is safely transferred to Statistics Sweden. Statistics Sweden will anonymise the data by replacing PIN with serial numbers. Data will be extracted from LISA (Longitudinal integration database for health insurance and labour market studies) and transferred to the entity principally responsible for the Swedish Osteoarthritis and Diabetes (SOAD) cohort research. The PIN and serial numbers will also be shared with National Board of Health and Welfare who will return data from National Patient Register (NPR), Swedish Prescribed Drug Register (SPDR), Cause of Death Register (CODR) and Swedish Cancer Register (SCR) to the entity principally responsible for the research. The linkage key will be saved at Statistics Sweden for 3 years to allow the possibility of adding more year cohorts or new variables to the research database if new research questions arise (with new ethical approval).

For peer review only

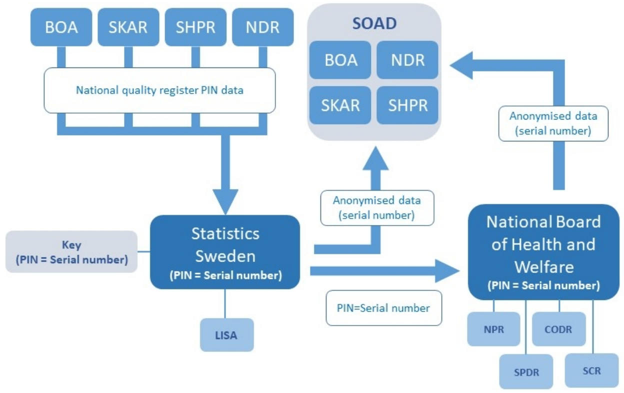

The data linkage process. Data from the four national quality registers Better Management of Patients with Osteoarthritis Register (BOA), Swedish Hip Arthroplasty Register (SHAR), Swedish Knee Arthroplasty Register (SKAR) and National Diabetes Register (NDR) is safely transferred to Statistics Sweden. Statistics Sweden will anonymise the data by replacing PIN with serial numbers. Data will be extracted from LISA (Longitudinal integration database for health insurance and labour market studies) and transferred to the entity principally responsible for the Swedish Osteoarthritis and Diabetes (SOAD) cohort research. The PIN and serial numbers will also be shared with National Board of Health and Welfare who will return data from National Patient Register (NPR), Swedish Prescribed Drug Register (SPDR), Cause of Death Register (CODR) and Swedish Cancer Register (SCR) to the entity principally responsible for the research. The linkage key will be saved at Statistics Sweden for 3 years to allow the possibility of adding more year cohorts or new variables to the research database if new research questions arise (with new ethical approval).

338x190mm (96 x 96 DPI)

STROBE Statement—checklist of items that should be included in reports of observational studies

	Item No.	Recommendation	Page No.	Relevant text from manuscript
**Title and abstract**	1	(*a*) Indicate the study's design with a commonly used term in the title or the abstract	1-2	
		(*b*) Provide in the abstract an informative and balanced summary of what was done and what was found	2	
**Introduction**				
Background/rationale	2	Explain the scientific background and rationale for the investigation being reported	4-5	
Objectives	3	State specific objectives, including any prespecified hypotheses	5	
**Methods**				
Study design	4	Present key elements of study design early in the paper	6-8	
Setting	5	Describe the setting, locations, and relevant dates, including periods of recruitment, exposure, follow-up, and data collection	6-8	
Participants	6	(*a*) *Cohort study*—Give the eligibility criteria, and the sources and methods of selection of participants. Describe methods of follow-up<br>*Case-control study*—Give the eligibility criteria, and the sources and methods of case ascertainment and control selection. Give the rationale for the choice of cases and controls<br>*Cross-sectional study*—Give the eligibility criteria, and the sources and methods of selection of participants	7-8	
		(*b*) *Cohort study*—For matched studies, give matching criteria and number of exposed and unexposed<br>*Case-control study*—For matched studies, give matching criteria and the number of controls per case		
Variables	7	Clearly define all outcomes, exposures, predictors, potential confounders, and effect modifiers. Give diagnostic criteria, if applicable	9, table 5	
Data sources/ measurement	8*	For each variable of interest, give sources of data and details of methods of assessment (measurement). Describe comparability of assessment methods if there is more than one group	Table 1 to 4	
Bias	9	Describe any efforts to address potential sources of bias	9	
Study size	10	Explain how the study size was arrived at	7-8 (BOA), 8 (other registers) 10	

For peer review only - http://bmjopen.bmj.com/site/about/guidelines.xhtml
For peer review only

			(sample size), Figure 1 (merging process)	
Quantitative variables	11	Explain how quantitative variables were handled in the analyses. If applicable, describe which groupings were chosen and why	9	
Statistical methods	12	(*a*) Describe all statistical methods, including those used to control for confounding	9	
		(*b*) Describe any methods used to examine subgroups and interactions	9	
		(*c*) Explain how missing data were addressed	9	
		(*d*) *Cohort study*—If applicable, explain how loss to follow-up was addressed<br>*Case-control study*—If applicable, explain how matching of cases and controls was addressed<br>*Cross-sectional study*—If applicable, describe analytical methods taking account of sampling strategy	9	
		(*e*) Describe any sensitivity analyses		
**Results**				
Participants	13*	(a) Report numbers of individuals at each stage of study—eg numbers potentially eligible, examined for eligibility, confirmed eligible, included in the study, completing follow-up, and analysed		N/A
		(b) Give reasons for non-participation at each stage		N/A
		(c) Consider use of a flow diagram		N/A
Descriptive data	14*	(a) Give characteristics of study participants (eg demographic, clinical, social) and information on exposures and potential confounders		N/A
		(b) Indicate number of participants with missing data for each variable of interest		N/A
		(c) *Cohort study*—Summarise follow-up time (eg, average and total amount)		N/A
Outcome data	15*	*Cohort study*—Report numbers of outcome events or summary measures over time		N/A
		Case-control study—Report numbers in each exposure category, or summary measures of exposure		N/A
		Cross-sectional study—Report numbers of outcome events or summary measures		N/A
Main results	16	(*a*) Give unadjusted estimates and, if applicable, confounder-adjusted estimates and their precision (eg, 95% confidence interval). Make clear which confounders were adjusted for and why they were included		N/A
		(*b*) Report category boundaries when continuous variables were categorized		N/A
		(*c*) If relevant, consider translating estimates of relative risk into absolute risk for a meaningful time period		N/A

Continued on next page

For peer review only - http://bmjopen.bmj.com/site/about/guidelines.xhtml

Other analyses	17	Report other analyses done—eg analyses of subgroups and interactions, and sensitivity analyses		N/A
**Discussion**				
Key results	18	Summarise key results with reference to study objectives		N/A
Limitations	19	Discuss limitations of the study, taking into account sources of potential bias or imprecision. Discuss both direction and magnitude of any potential bias		N/A
Interpretation	20	Give a cautious overall interpretation of results considering objectives, limitations, multiplicity of analyses, results from similar studies, and other relevant evidence		N/A
Generalisability	21	Discuss the generalisability (external validity) of the study results		N/A
**Other information**				
Funding	22	Give the source of funding and the role of the funders for the present study and, if applicable, for the original study on which the present article is based	14	

*Give information separately for cases and controls in case-control studies and, if applicable, for exposed and unexposed groups in cohort and cross-sectional studies.

Note: An Explanation and Elaboration article discusses each checklist item and gives methodological background and published examples of transparent reporting. The STROBE checklist is best used in conjunction with this article (freely available on the Web sites of PLoS Medicine at http://www.plosmedicine.org/, Annals of Internal Medicine at http://www.annals.org/, and Epidemiology at http://www.epidem.com/). Information on the STROBE Initiative is available at www.strobe-statement.org.

For peer review only
